# Analysis of Energy Metabolism and Lipid Spatial Distribution in Hypoxic-Ischemic Encephalopathy Revealed by MALDI-MSI

**DOI:** 10.3390/biomedicines13061431

**Published:** 2025-06-11

**Authors:** Xingxing Zhao, Peipei Chen, Lun Yu, Chuchu Gao, Sannan Wang, Zuming Yang, Zongtai Feng

**Affiliations:** Department of Neonatology, The Affiliated Suzhou Hospital of Nanjing Medical University (Suzhou Municipal Hospital), No. 26, Daofront Street, Suzhou 215002, China; xxzhao2018@163.com (X.Z.); 18068425020@163.com (P.C.); lovely_taotao@163.com (L.Y.); chuchugao1987@163.com (C.G.); wangsn2002@sina.com (S.W.)

**Keywords:** neonatal hypoxic-ischemic encephalopathy, spatial metabolome, energy metabolism, purine metabolism, lipid metabolism

## Abstract

**Background:** Neonatal hypoxic-ischemic encephalopathy (HIE) is a major cause of neonatal death and neurodevelopmental disorders, and its pathological mechanisms are closely related to disturbed energy metabolism and lipid remodeling. Exploring the spatial heterogeneity of metabolomics is essential to analyze the pathological process of HIE. **Methods:** In this study, we established a neonatal mouse hypoxic-ischemic brain damage (HIBD) model by the modified Rice method, and analyzed various metabolic pathways such as the tricarboxylic acid (TCA) cycle, purine metabolism, and lipid metabolism in the ischemic edema area, with contralateral and control brain tissues using matrix-assisted laser desorption mass spectrometry imaging (MALDI-MSI) with a spatial resolution of 50 μm. **Results:** In the HIBD model, key metabolites of the tricarboxylic acid (TCA) cycle (citrate, succinate, L-glutamate, glucose, aspartate, and glutamine) were significantly enriched in the edematous area compared with the control (fold change: 1.52–2.82), which suggests a blockage of mitochondrial function; ATP/ADP/AMP levels were reduced by 53–73% in the edematous area, and xanthine was abnormally accumulated in the hippocampus of the affected side, suggesting energy depletion and altered purine metabolism; lipid remodeling showed regional specificity: some unsaturated fatty acids, such as docosahexaenoic acid, were abnormally accumulated in the hippocampus. In contrast, pentadecanoic acid levels were reduced across the entire brain in the HIBD model, with a more pronounced decrease in the ipsilateral hippocampus, suggesting impaired membrane stability. **Conclusions:** The neonatal mouse HIBD model exhibits reprogramming of energy metabolism, characterized by a blockage in the tricarboxylic acid (TCA) cycle and ATP depletion, along with an abnormal spatial distribution of lipids. By targeting xanthine metabolic pathways, restoring mitochondrial function, and intervening in region-specific lipid remodeling, brain energy homeostasis may be improved and neurological damage attenuated. Further studies should validate the clinical feasibility of xanthine and lipid imbalance as diagnostic markers of HIBD and explore the critical time window for metabolic intervention to optimize therapeutic strategies.

## 1. Introduction

Neonatal hypoxic-ischemic encephalopathy (HIE) is a severe neurological injury caused by perinatal hypoxia and ischemia of brain tissue [1], and is one of the leading causes of neonatal mortality and neurological deficits in children worldwide. The annual incidence of HIE is about 1–3‰ [2], of which about 25–50% of children die within two weeks of the onset of the disease, and 40–60% die or have severe disabilities before the age of two years. Deaths due to HIE account for 22% of global neonatal deaths [3], and 25–30% of survivors have residual neurological disabilities (e.g., cerebral palsy, cognitive impairments) [4]. Early diagnosis and intervention are essential to improve the prognosis, but there is a lack of biomarkers with high sensitivity and specificity, and precise assessment tools.

In recent years, studies targeting the pathological mechanisms of HIE have focused on the exploration of molecular markers. Through techniques such as enzyme-linked immunosorbent assay (ELISA) and protein blotting (Western blot), successive researchers have found that protein markers such as IL-6 and TNF-α are associated with the course of HIE [5]. However, these markers are difficult to meet clinical needs due to insufficient sensitivity, cumbersome operation, or limited information in a single test. The development of metabolomics technology has provided new perspectives for HIE research: studies based on nuclear magnetic resonance (1H NMR), gas chromatography-mass spectrometry (GC-MS), and other methods have revealed disturbances in amino acid metabolism, energy metabolism, and oxidative stress pathways in the course of HIE. For example, elevated urinary lactate can serve as an early warning indicator for high-risk children [6], whereas dynamic changes in plasma substances such as Tau and BDNF significantly correlate with the degree of neurological damage [7,8], and elevated levels of serum neuron-specific enolase and S100 calcium-binding protein B are associated with a poorer prognosis in neonates with perinatal asphyxia [9]. Nevertheless, traditional metabolomics techniques are unable to resolve the spatial distribution characteristics of metabolites, and the heterogeneity of brain tissues makes the dynamic changes in the local metabolic microenvironment (e.g., hippocampal region, cortical edema region) key to understanding the mechanisms of HIE.

**Figure 1:**
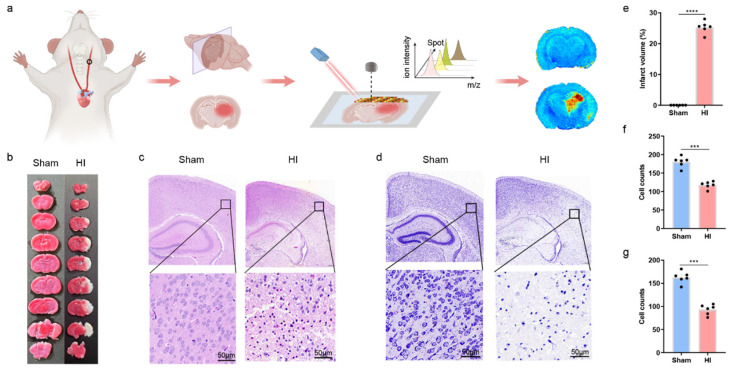
Pathology and spatial metabolomics process of HIE mouse model. (**a**) Graphic experimental procedures in this study. Coronal sections were obtained from sham and HI (hypoxic-ischemic) mouse brains, then scanned and analyzed using MALDI-MSI. Metabolite features were extracted and identified for further analysis. (**b**) TTC staining detected the infarct size in sham and HI groups. (**c**) H&E staining of brain sections from sham and HI groups was shown. (**d**) Nissl staining of cortical tissue in sham and HI groups. (**e**) Quantification of brain infarct size in sham and HI groups. (**f**) Quantitative assessment of the number of viable neurons/200× field were recorded in each group. (**g**) Quantitative assessment of the number of cells in d. Data were presented by Mean ± SD, *** *p* < 0.001, **** *p* < 0.0001.

The relatively recent matrix-assisted laser desorption ionization mass spectrometry imaging (MALDI-MSI) technique, which has emerged as a powerful tool in biomarker research, enables in situ visualization of metabolites through micrometer spatial resolution [10], providing a new perspective for revealing metabolic heterogeneity in HIE. Currently, studies have reported the association of TCA circulatory metabolic abnormalities with energy failure in HIE [11], but the spatial and temporal characteristics of lipid remodeling in specific brain regions (e.g., hippocampus, cortical edema areas) still lack systematic resolution. In this study, we constructed a neonatal mouse hypoxic-ischemic brain damage (HIBD) model based on the improved Rice method [12], integrated MALDI-MSI technology to analyze the spatial metabolic reprogramming characteristics of brain tissues, and systematically resolved the spatial distribution pattern of small-molecule metabolites and pathway changes of brain tissues in the process of HIE (Figure 1a). By comparing the metabolic profiles of edematous, contralateral, and control regions, we focused on (1) the spatial gradient distribution of key metabolites (citrate, succinate) and glutamatergic pathways in the TCA cycle; (2) the dynamic association between imbalance of ATP metabolism and purine degradation products; and (3) the molecular mechanism of hippocampus-specific lipid remodeling (e.g., docosahexaenoic acid accumulation, pentadecanoic acid down-regulation) and membrane stability impairment.

## 2. Materials and Methods

### 2.1. Animals and HIBD Models

Ten-day-old C57BL/6 mice were procured from Hangzhou ziyuan Experimental Animal Technology Co., Ltd. (Hangzhou, China). The neonatal mice were nursed by their mothers and were kept at an ambient temperature of 25 ± 2 °C under a controlled 12 h light/dark cycle. All protocols for the in vivo experiments were approved by the Animal Experiment Ethics Committee.

A total of 18 neonatal mice (10-day-old C57BL/6) were used in the study, randomly divided into two groups: hypoxic-ischemic (HI) model group and sham control group, with 9 mice in each group. Of these, 6 mice per group were used for TTC staining, H&E, and Nissl staining analyses, and 3 mice per group were used for MALDI-MSI and subsequent metabolomic and pathological imaging analyses.

The HIBD model was established according to a modified Rice method. Briefly, a newborn (10-day-old neonatal) mouse was anesthetized with isoflurane before the left common carotid artery was separated and ligated; the skin was then sutured. After recovering beside the mother mouse for 1 h, the pups were placed in an oxygen-deficient chamber (8% O_2_ and 92% N_2_) at 37 °C for 2 h before being returned to normal conditions. Take brain tissue 24 h after modeling.

### 2.2. TTC Staining

The infarct area was measured by TTC staining (Solarbio, Beijing, China) 24 h after HIBD modeling. Briefly, mouse brains were sliced into 1–2 mm thick sections using the coronal brain matrices (ASI Instruments, Warren, MI, USA), stained at 37 °C for 10–15 min protected from light, and then immersed in 4% paraformaldehyde solution for 6 h for subsequent quantitative photography.

### 2.3. MALDI Mass Spectrometry Imaging and Data Analysis

Brain sections were vacuum-dried before tissue marking and high-resolution scanning (7200 dpi) on ITO slides. A homogeneous matrix coating was deposited using electric field-assisted spray technology, employing a 50% ethanol solution containing 4.39 mg/mL 1,5-DAN hydrochloride and 55 mM HCl (250 μL total volume).

MSI analysis was conducted on a rapifleX MALDI-TOF/TOF system (Bruker Daltonics, Bremen, Germany) with a 10 kHz smartbeam 3D laser in negative ion mode. Instrument parameters included 50% laser power (10% offset), 11.35 kV lens voltage, and 20.85 kV reflector voltage. Spatial resolution was maintained at either 50 μm or 20 μm across the *m*/*z* 100–1200 range, collecting 100 laser shots per spectrum. Data processing utilized SCiLS Lab 2023c (Bruker, Bremen, Germany) with total ion current normalization for subsequent analytical workflows.

### 2.4. Statistical Analysis

Data were analyzed using GraphPad Prism 9 and the results are expressed as the mean standard deviation (S.D). To make sure the data were normal, the Shapiro–Wilk test was run. To find statistically significant differences between the groups, post hoc Tukey’s tests and one- or two-way analysis of variance (ANOVA) were used. Statistical significance was determined at *p* < 0.05 for all comparisons.

## 3. Results

In order to verify the establishment of the HIBD model, tissues were stained with TTC, hematoxylin and eosin (HE), and Nissl staining to observe the pathological changes. TTC staining showed that the ipsilateral hemisphere of HI mice exhibited obvious infarction and swelling, in contrast to the symmetrical appearance of the sham group (Figure 1b,e). The affected hemisphere appeared enlarged due to acute brain edema following carotid artery ligation. The HE staining showed that there were large infarcts, and the number of cells in the infarcts was reduced (Figure 1c,f). Nissl staining showed that the nerve tissue damage was more obvious in the HI group than in the Sham group (Figure 1d,g). These histological changes confirmed the establishment of the HIBD model. Using 1,5-DANHCl as a substrate, the identified metabolites are summarized in Table 1.

### 3.1. Blockage of the TCA Cycle

**Figure 2:**
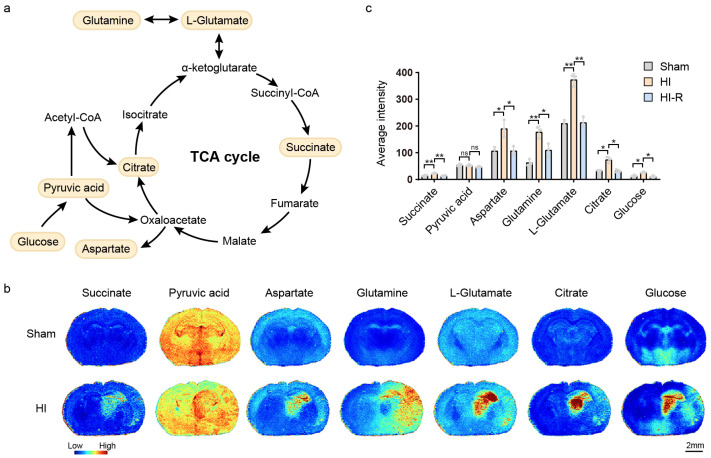
TCA cycle MALDI mass spectrometry imaging. (**a**) Simplified overview of TCA cycle. (**b**) MALDI MS images of seven metabolites in TCA cycle. (**c**) Average intensities of seven metabolites in ischemic core region (HI), contralateral region of the modeled mice (HI-R), and ipsilateral side of the control mice (Sham). Scale bar, 2 mm. Data are shown as the mean ± SD (n = 3), * *p* < 0.05, ** *p* < 0.01, ns indicates that there is no significant difference.

The TCA cycle is a central pathway of cellular energy metabolism, beginning with the conversion of glucose to acetyl coenzyme A via glycolysis-generated pyruvic acid, followed by condensation with oxaloacetate to form citrate, which undergoes decarboxylation and oxidation to produce succinate, accompanied by the production of reducing equivalents (NADH/FADH_2_). Glutamate and aspartate are involved in the regeneration of intermediates (e.g., α-ketoglutarate, oxaloacetic acid) through transamination, whereas glutamine can indirectly maintain TCA cyclic flux by deaminating to produce glutamate, which collectively drives the dynamic balance between ATP synthesis and carbon skeleton metabolism (Figure 2a). The TCA cycle is disrupted under conditions of ischemia and hypoxia [13]. The accumulation of tricarboxylic acid cycle-related metabolites in the ischemic core region is shown in Figure 2b,c. Compared with the contralateral region of the modeled mice and with the ipsilateral side of the control mice, the mean relative amounts of glucose increased 2.35-fold and 1.92-fold, respectively, Citrate increased 2.33-fold and 2.16-fold, respectively, and L-glutamate increased 175-fold and 1.78-fold, respectively. Glutamine increased 1.62-fold and 2.82-fold, succinate increased 1.60-fold and 1.52-fold, and aspartate increased 1.77-fold and 1.78-fold, respectively, with glutamine elevated throughout the brain of the modeled mice compared to the control mice, most notably in the cortex. Interestingly, pyruvic acid was not significantly different in the ischemic core region of modeled and control mice, but was decreased in the contralateral region of modeled mice compared with the modeled side. These abnormalities suggest a significant blockade of the tricarboxylic acid cycle under ischemic hypoxic conditions. The significant enrichment of glucose, which may reflect enhanced compensatory glycolysis, was not accompanied by a concomitant increase in pyruvic acid (no difference between the modeled side and the control), suggesting that its conversion to lactate or mitochondrial pyruvate transport is blocked; the concomitant accumulation of citrate and succinate suggests that the inhibition of mitochondrial complex I (NADH dehydrogenase) and complex II (succinate dehydrogenase) activities leads to the disruption of the electron transport chain and the blockage of the TCA cycle in the mitochondria. Pyruvic acid is reduced in regions contralateral to modeling, suggesting compensatory energy metabolic redistribution, possibly through lactate shunting or alanine production to maintain acid-base balance.

### 3.2. Impairment of Purine Metabolism

**Figure 3:**
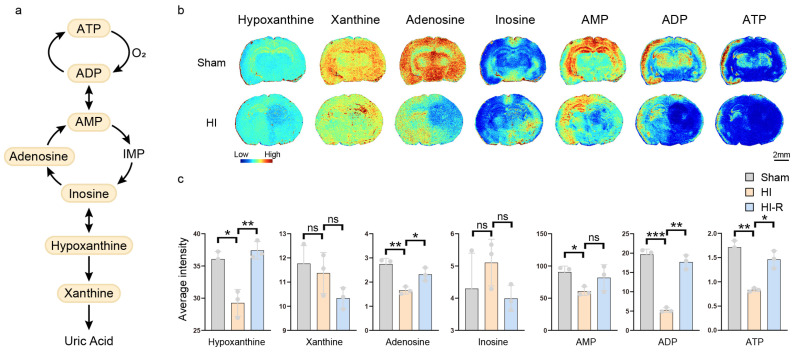
Purine metabolism MALDI mass spectrometry imaging. (**a**) Simplified overview of purine metabolism. (**b**) MALDI MS images of seven metabolites in purine metabolism. (**c**) Average intensities of seven metabolites in ischemic core region (HI), contralateral region of the modeled mice (HI-R), and ipsilateral side of the control mice (Sham). Scale bar, 2 mm. Data are shown as the mean ± SD (n = 3), * *p* < 0.05, ** *p* < 0.01, *** *p* < 0.001, ns indicates that there is no significant difference.

Purine metabolism is the core pathway to maintain cellular energy homeostasis and nucleotide synthesis, and its core links include: (1) purine nucleotide synthesis: with inosine monophosphate (IMP) as the pivotal point, it is generated by branching pathway to adenosine monophosphate (AMP) and guanosine monophosphate (GMP), which is further phosphorylated to ATP/GTP; (2) purine degradation: ATP is generated by gradual dephosphorylation to adenosine diphosphate (ADP), AMP and adenosine, which is ultimately catalyzed by deaminase and xanthine oxidase to hypoxanthine, xanthine, and uric acid (Figure 3a). This process is highly dependent on the partial pressure of oxygen, as oxygen is required as an electron acceptor for xanthine oxidase activity [14]. Purine metabolism was characterized by a significant imbalance under ischemic hypoxic conditions. In the ischemic core region compared with the contralateral region of modeled mice and compared with the ipsilateral side of control mice, the mean relative amount of ATP was reduced by 43% and 51%, ATP by 70% and 73%, AMP by 26% and 33%, and adenosine by 29% and 40%, these changes are suggestive of a breakdown in mitochondrial oxidative phosphorylation function leading to disruption of ATP synthesis with compensatory acceleration of nucleotide degradation to transiently sustain cellular energy requirements. Inosine increased 1.28-fold and 1.19-fold, hypoxanthine decreased 22% and 19%, and xanthine was somewhat enriched (1.1-fold) compared with the contralateral side, suggesting that the conversion of adenosine to inosine is enhanced to delay energy depletion. In addition, GMP, IMP, and UMP were all significantly reduced in abundance in the ischemic core region compared with the contralateral region of the modeled mice, by 56%, 70%, and 53%, respectively, and GMP and IMP were elevated in the contralateral region of the modeled mice compared with the same region of the control mice (Figure 3b,c). This may be due to the possibility that the contralateral region partially compensates for the energy deficit and maintains nucleic acid synthesis by activating purine remediation synthesis pathways (e.g., hypoxanthine-guanine phosphoribosyltransferase (HGPRT)) [15], which utilize the degradation products, such as hypoxanthine, to re-synthesize IMP, which in turn generates GMP and ATP.

### 3.3. Alterations in Unsaturated Fatty Acid Metabolism

Unsaturated fatty acid metabolism is characterized by the conversion of linoleic acid and α-linolenic acid to arachidonic acid and docosahexaenoic acid (core component of the neuromembrane structure) catalyzed by the enzymes Δ-6 desaturase and elongase, and by the conversion of oleic acid from stearic acid by Δ-9 desaturase, which is involved in the energy metabolism of unsaturated fatty acids. Oleic acid, converted from stearic acid by Δ-9 desaturase, is involved in energy storage and regulation of membrane fluidity.

**Figure 4:**
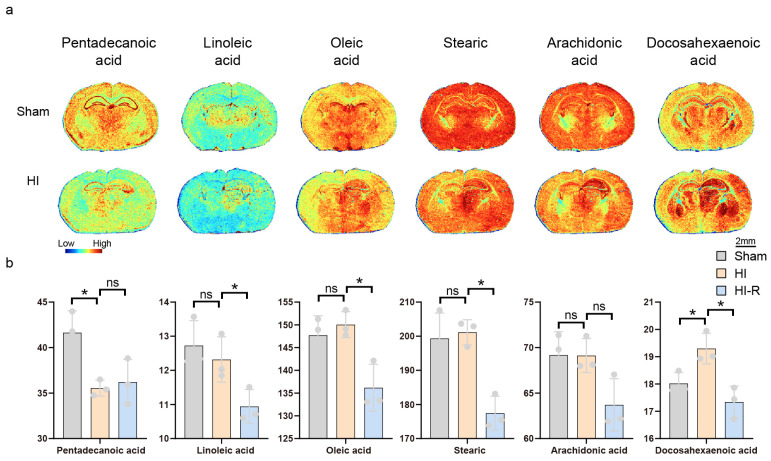
MALDI mass spectrometry imaging and Statistical Chart of Unsaturated Fatty Acid Metabolism. (**a**) MALDI MS images of six unsaturated fatty acids. (**b**) Average intensities of six unsaturated fatty acids in ischemic core region (HI), contralateral region of the modeled mice (HI-R), and ipsilateral side of the control mice (Sham). Scale bar, 2 mm. Data are shown as the mean ± SD (n = 3), * *p* < 0.05, ns indicates that there is no significant difference.

The mean relative amounts of linoleic acid and oleic acid were slightly elevated in the ischemic core region compared to the contralateral region of the modeled mice. The elevation of oleic acid as a monounsaturated fatty acid (ω-9) may be related to the compensatory regulation of membrane fluidity in response to cellular stress, whereas the accumulation of linoleic acid (ω-6) may originate from the metabolic blockade caused by the inhibition of Δ-6 desaturase activity. Inhibition of Δ-6 desaturase activity leads to metabolic blockage, suggesting an imbalance in ω-6/ω-3 metabolism. Arachidonic acid, as a precursor of eicosanoids, is elevated, suggesting ischemia-induced activation of phospholipase A2 (PLA2), resulting in the catabolism of membrane phospholipids and release of free fatty acids, which initiates the synthesis of pro-inflammatory mediators. The mild increase in stearic acid may reflect the decrease in mitochondrial β-oxidation capacity, the failure of saturated fatty acids to be decomposed to acetyl coenzyme A, or the restricted activity of desaturase (e.g., SCD1), which results in the blockage of their conversion to oleic acid; while there was a significant decrease in pentadecanoic acid, which, as an odd-chain fatty acid, is decomposed to propionyl coenzyme A by α-oxidation, and the decrease suggests the dysfunction of mitochondrial β-oxidation (Figure 4a,b).

### 3.4. Phosphoglyceride and Other Metabolite Alterations

**Figure 5:**
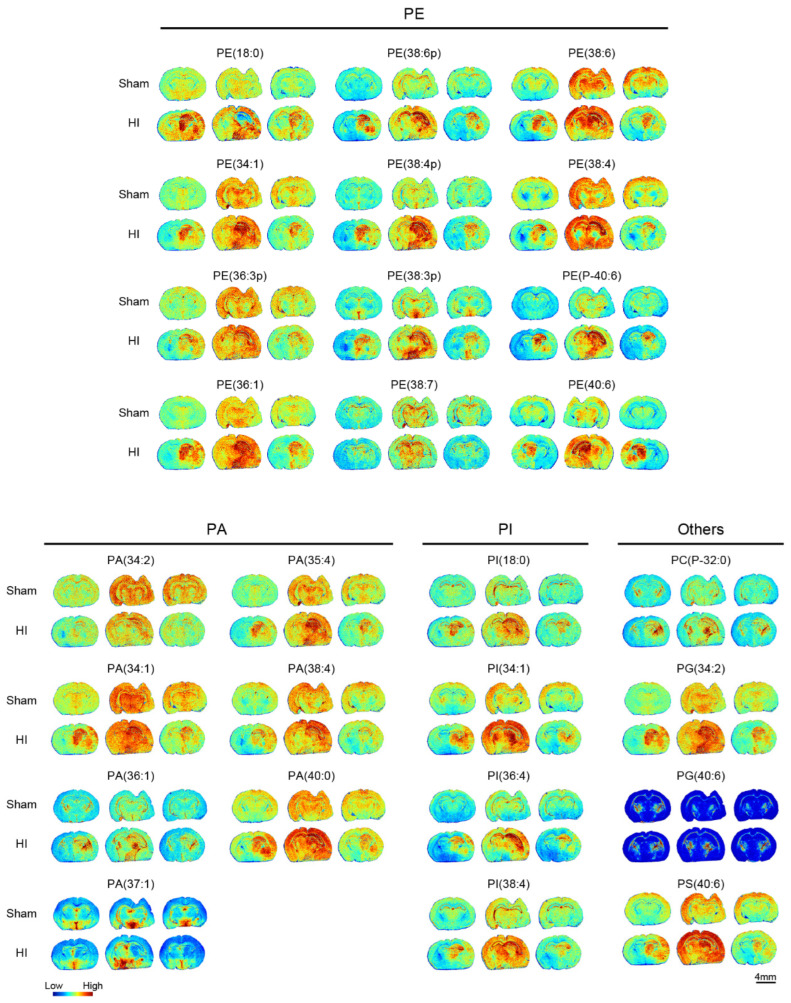
MALDI mass spectrometry imaging of lipids in sham and Hl groups (n = 3). Six types of lipids were detected, including 12 PEs (phosphatidylethanolamine), 7 PAs (phosphatidic acid), 4 PIs (phosphatidylinositol), and other lipids. Scale bar, 4 mm.

Lipid metabolism is a core process to maintain the dynamic balance of cell membranes and signaling, in which phosphatidic acid (PA), as a key precursor of phospholipid synthesis, participates in membrane structural remodeling by regulating downstream phosphatidylethanolamine (PE) and phosphatidylinositol (PI) generation; PE is one of the major components of cell membranes, affecting membrane fluidity and autophagic vesicle formation, whereas PI is generated through phosphorylation to generate PIP2 and PIP3, and other signaling molecules through phosphorylation, which mediate cell proliferation, apoptosis, and other key pathways. Abnormal metabolism of the three can lead to membrane dysfunction and signaling network disorders, which are closely related to neurodegenerative diseases and cancer. Our data demonstrated alterations of 27 phosphoglycerides in the neonatal mouse HIBD model (Figure 5), most of which accumulated to some extent in the ischemic core region (e.g., PE38:6p was 1.43-fold higher than the average abundance in the contralateral region of the modeled mice, whereas the abundance of PA (37:1) was reduced to some extent). Such changes may reflect an imbalance between energy metabolism and membrane repair in neuronal membrane structures.

**Figure 6:**
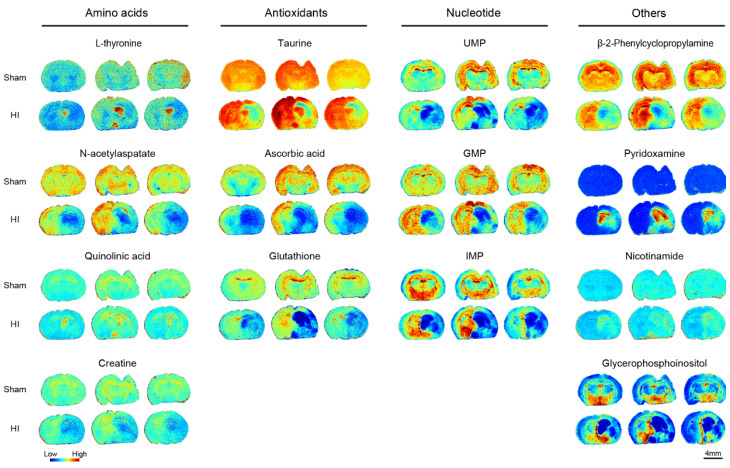
MALDI mass spectrometry imaging of other metabolites in sham and HI groups (n = 3). Fourteen other metabolites were detected, mainly consisting of four amino acids, three antioxidants, and three nucleotides. The other 4 metabolites were β-2-phenylamine, pyridoxamine, nicotinamide, and glycerophosphoinositol. Scale bar, 4 mm.

In addition, our data also demonstrated changes in the abundance of amino acids of some other metabolites such as antioxidants (Figure 6). Interestingly, ascorbic acid, taurine, and glutathione all showed significant reductions in the ischemic core region. Compared to the contralateral side of the modeled mice, their average abundances were reduced by 54%, 26%, and 51%, respectively. When compared to the ipsilateral side of the control mice, the reductions were 62%, 13%, and 54%, respectively. These changes suggest that bursts of reactive oxygen species (ROS) in the ischemic region may deplete antioxidant systems—particularly glutathione—thereby triggering mitochondrial dysfunction and a lipid peroxidation chain reaction, ultimately exacerbating neuronal apoptosis. In addition, N-acetylaspatate, creatine, β-2-phenylcyclopropylamine, and glycerophosphoinositol showed significant decreases, reflecting impaired neuronal mitochondrial function or neuronal loss, deficient ATP regeneration capacity, and abnormal membrane phospholipid metabolism. In contrast, nicotinamide and pyridoxamine significantly accumulated in the ischemic core region, possibly reflecting cellular attempts to resist ischemic injury through reprogramming of vitamin metabolism. To support the spatial visualization results, the original MALDI-MSI signal intensities (mean values across three biological replicates per group and region) are presented in Supplementary Table S1.

## 4. Discussion

Our study spatially resolved metabolic reprogramming in a neonatal mouse HIBD model using MALDI-MSI, providing new insights into the heterogeneous alterations in energy and lipid metabolism in hypoxic-ischemic brain injury. Spatial accumulation of TCA circulating intermediates (e.g., citrate, succinate) in the ischemic edema zone suggests severe mitochondrial dysfunction. Whereas glycolysis is normally upregulated in hypoxia to compensate for ATP deficiency [16], we observed elevated glucose and relatively insignificant changes in pyruvate levels, suggesting impaired pyruvate entry into the mitochondria or preferential shunting of pyruvate to lactate production. This is consistent with previous studies showing that hypoxia inhibits pyruvate dehydrogenase, a key enzyme linking glycolysis to the TCA cycle [17]. The concomitant enrichment of citrate and succinate further suggests disruption of the electron transport chain (ETC), which may be due to inhibition of mitochondrial complexes I and II, as reported in ischemic stroke models [18]. Accumulation of glutamine in the HIBD brain suggests the presence of a compensatory anaerobic flux to replenish the TCA intermediates, which does not rescue the depletion of ATP. These results suggest that HIE energy depletion is accompanied by mitochondrial ETC dysfunction, suggesting the possibility of developing therapies that target mitochondrial protection or alternative energy substrate delivery.

Notably, our findings show remarkable consistency with recent MALDI-MSI-based studies, which similarly reported the accumulation of glucose, citric acid, and glutamine in the TCA cycle, as well as marked depletion of ATP and concomitant increases in xanthine and inosine within ischemic brain regions [19]. Comparable alterations in taurine and creatine levels were also observed [20], underscoring disruptions in osmolyte balance and energy-buffering mechanisms. Together, these parallels point to a conserved metabolic signature of mitochondrial dysfunction and energy failure across various models of cerebral ischemia.

The dramatic decrease (>50%) in ATP/ADP/AMP levels in the ischemic core reflects a breakdown in oxidative phosphorylation, forcing the cell to rely on purine degradation for temporary energy replenishment. The accumulation of xanthine in the hippocampus may be the result of hypoxia-triggered activation of xanthine oxidase, a phenomenon associated with oxidative stress in neonatal HIE [21]. Interestingly, the contralateral region exhibited elevated IMP and GMP, suggesting the possible existence of compensatory purine salvage pathways (e.g., HGPRT-mediated hypoxanthine cycle [15]). However, this adaptation was not sufficient to counteract overall ATP depletion, especially in the ischemic core. These findings suggest that purine degradation products (e.g., uric acid) may serve as biomarkers of HIE severity and suggest that modulation of xanthine oxidase activity or enhancement of the purine cycle may alleviate energy depletion. Importantly, the potential therapeutic implications of modulating the xanthine oxidase pathway have been increasingly recognized. Allopurinol, a well-established xanthine oxidase inhibitor, has shown promise in preclinical models of neonatal HIE by reducing oxidative stress and improving neurological outcomes. Clinical studies have demonstrated that early administration of allopurinol may attenuate brain injury when used alone or in combination with therapeutic hypothermia. A comprehensive review by Annink et al. [22] summarized existing evidence supporting its safety and efficacy. Furthermore, the ongoing ALBINO trial (NCT03162653) [23] is evaluating the clinical utility of allopurinol in asphyxiated newborns, aiming to clarify its neuroprotective potential in a well-powered, randomized setting. These findings underscore the translational relevance of our observation of xanthine accumulation in ischemic brain regions, and support the exploration of xanthine oxidase inhibition as a therapeutic strategy in HIE.

The region-specific lipid alterations we observed reveal complex metabolic adaptations and vulnerabilities. Accumulation of hippocampal docosahexaenoic acid (DHA), a key component of neuronal membranes, contrasts with the rapid depletion typical during ischemia, which may reflect impaired membrane repair or utilization of antioxidant responses. In contrast, an overall reduction in pentadecanoic acid, an odd-chain fatty acid metabolized via β-oxidation, highlighted the impairment of mitochondrial β-oxidation [24], which is consistent with disruption of circulating fluxes of TCA. Meanwhile, the increase in arachidonic acid suggests activation of phospholipase A2 and synthesis of proinflammatory eicosanoids [25], which may exacerbate neuroinflammation. These lipid alterations emphasize the dual role of lipid metabolism in HIE: while certain lipids (e.g., DHA) may initially protect cell membranes, their eventual dysregulation leads to oxidative stress and neuronal death. Targeting region-specific lipid pathways, such as enhancing DHA supplementation or inhibiting arachidonic acid-derived inflammatory mediators, warrants further exploration.

Antioxidants (e.g., glutathione, ascorbic acid) are drastically decreased in ischemic regions, which is consistent with previous reports that oxidative stress is a hallmark of HIE [26]. Glutathione depletion may exacerbate mitochondrial damage and lipid peroxidation [27], resulting in a vicious cycle of neuronal damage. Furthermore, the accumulation of nicotinamide and pyridoxamine suggests endogenous attempts to counteract oxidative stress through vitamin-mediated pathways [28]. This highlights the potential of antioxidant therapies, although their efficacy may depend on precise timing and delivery to affected regions.

Although our study provides high-resolution spatial metabolomics data, there are several limitations that must be addressed. First, the HIBD model captures metabolic changes at 24 h post-injury, which represents a relatively late stage of the early response [29,30,31]. As clinical HIE evolves dynamically, the most critical window for intervention may occur during reperfusion and prior to the development of secondary injury. Future studies should include earlier time points (e.g., 1–6 h post-HIBD) to better characterize the initial metabolic shifts and assess the potential for prophylactic intervention before irreversible damage occurs. Second, the clinical translational power of xanthine or lipid biomarkers requires more experimental validation. Finally, testing whether modulation of specific pathways (e.g., inhibition of xanthine oxidase, DHA supplementation) rescues metabolic defects and improves prognosis requires mechanistic studies. Furthermore, we acknowledge the limited sample size (n = 3 per group) for MALDI-MSI-based analyses. Expanding sample sizes in future work would enhance statistical power and the generalizability of spatial metabolomic trends.

## 5. Conclusions

By combining MALDI-MSI with spatial metabolomics, the present study revealed a metabolic profile of HIBD in mice characterized by mitochondrial failure, purine degradation, and region-specific lipid dysregulation. These findings position xanthine metabolism, mitochondrial protection, and lipid remodeling as promising therapeutic targets. Future work should focus on translating these insights into clinically feasible strategies to restore energy homeostasis and attenuate neurologic sequelae in HIE.

## Figures and Tables

**Table 1:** Metabolites identified using 1,5-DANHCl as a substrate.

Name	Theoretical	Parent Ion	Experimental *m*/*z*	Delta (Da)	ppm
Succinate	117.018	[M−H]^−^	117.018	0.000	−2.008
Pyruvic acid	122.984	[M+Cl]^−^	122.984	0.000	−2.830
Taurine	124.007	[M−H]^−^	124.008	0.001	5.403
Creatine	130.061	[M−H]^−^	130.061	0.000	−0.792
Aspartate	132.030	[M−H]^−^	132.031	0.001	6.211
Hypoxanthine	135.031	[M−H]^−^	135.032	0.001	6.073
Glutamine	145.062	[M−H]^−^	145.063	0.001	8.203
L-Glutamate	146.046	[M−H]^−^	146.047	0.001	8.011
Xanthine	151.026	[M−H]^−^	151.026	0.000	−0.662
Nicotinamide	157.016	[M+Cl]^−^	156.952	−0.064	−409.620
Quinolinic acid	166.013	[M−H]^−^	166.015	0.002	9.132
Pyridoxamine	167.082	[M−H]^−^	167.080	−0.002	−9.002
β-Glycerophosphoric acid	171.005	[M−H]^−^	170.935	−0.070	−411.104
N-acetylaspartate	174.041	[M−H]^−^	174.042	0.001	7.240
Ascorbic acid	175.025	[M−H]^−^	175.026	0.001	7.085
Citrate	191.020	[M−H]^−^	191.021	0.001	6.963
Glucose	215.032	[M+Cl]^−^	215.033	0.001	6.083
Pentadecanoic acid	241.216	[M−H]^−^	241.218	0.002	7.433
Adenosine	266.088	[M−H]^−^	265.957	−0.131	−493.746
Inosine	267.073	[M−H]^−^	267.100	0.027	99.448
L-Thyronine	272.092	[M−H]^−^	272.031	−0.061	−223.211
Linoleic acid	279.232	[M−H]^−^	279.243	0.011	39.906
Oleic acid	281.248	[M−H]^−^	281.258	0.010	37.309
Stearic acid	283.263	[M−H]^−^	283.280	0.017	59.461
Arachidonic acid	303.232	[M−H]^−^	303.261	0.029	96.108
Glutathione	306.076	[M−H]^−^	306.136	0.060	194.461
Uridine monophosphate (UMP)	323.027	[M−H]^−^	323.055	0.028	85.154
Docosahexaenoic acid	327.232	[M−H]^−^	327.270	0.038	116.563
AMP	346.056	[M−H]^−^	346.079	0.023	67.186
Inosine-5′-monophosphate (IMP)	347.039	[M−H]^−^	347.110	0.071	205.378
GMP	362.051	[M−H]^−^	362.084	0.033	92.059
Glycerophosphoinositol	369.035	[M+Cl]^−^	369.065	0.030	81.830
ADP	426.022	[M−H]^−^	426.088	0.066	154.734
PE (18:0)	480.310	[M−H]^−^	480.382	0.072	150.924
ATP	505.988	[M−H]^−^	505.983	−0.005	−10.712
PI (18:0)	599.320	[M−H]^−^	599.369	0.049	81.542
NADH	664.116	[M−H]^−^	664.414	0.298	448.117
PA (34:2)	671.465	[M−H]^−^	671.501	0.036	54.162
PA (34:1)	673.481	[M−H]^−^	673.515	0.034	49.994
PC (P-16:0/P-16:0)	700.564	[M−H]^−^	700.570	0.006	8.633
PA (36:1)	701.513	[M−H]^−^	701.527	0.014	20.484
PA (22:1/15:0)	715.527	[M−H]^−^	715.627	0.100	139.433
PE (34:1)	716.524	[M−H]^−^	716.546	0.022	31.374
PA (15:0/20:4)	717.426	[M+Cl]^−^	717.569	0.143	199.798
PA (38:4)	723.497	[M−H]^−^	723.506	0.009	12.467
PE (36:3p)	724.528	[M−H]^−^	724.563	0.035	48.905
PE (36:1)	744.554	[M−H]^−^	744.635	0.081	109.083
PG 34:2 (18:2_16:0)	745.501	[M−H]^−^	745.606	0.105	140.292
PE (38:6p)	746.512	[M−H]^−^	746.576	0.064	85.843
PA (40:6)	747.497	[M−H]^−^	747.521	0.024	32.134
PE (38:4p)	750.543	[M−H]^−^	750.606	0.063	83.650
PE (38:3p)	752.559	[M−H]^−^	752.583	0.024	32.068
PE (38:7)	760.491	[M−H]^−^	760.582	0.091	119.422
PE (38:6)	762.508	[M−H]^−^	762.531	0.023	30.334
PE (38:4)	766.538	[M−H]^−^	766.542	0.004	5.047
PE (P-40:6)	774.544	[M−H]^−^	774.570	0.026	33.232
PE (40:6)	790.539	[M−H]^−^	790.563	0.024	30.144
PG (40:6)	821.533	[M−H]^−^	821.536	0.003	4.002
PS (40:6)	834.529	[M−H]^−^	834.563	0.034	40.742
PI (34:1)	835.533	[M−H]^−^	835.547	0.014	16.629
PI (36:4)	857.519	[M−H]^−^	857.545	0.026	30.903
PI (38:4)	885.550	[M−H]^−^	885.570	0.020	22.811

## Data Availability

All data used to support this study are available from the corresponding author upon request.

## Supplementary Materials

The following supporting information can be downloaded at: https://www.mdpi.com/article/10.3390/biomedicines13061431/s1, Table S1: Raw MALDI-MSI signal intensities (mean values) of selected lipid and antioxidant metabolites shown in Figure 5 and Figure 6 across ischemic, contralateral, and sham brain regions (n = 3 per group).
